# Functionally Cloned *pdrM* from *Streptococcus pneumoniae* Encodes a Na^+^ Coupled Multidrug Efflux Pump

**DOI:** 10.1371/journal.pone.0059525

**Published:** 2013-03-26

**Authors:** Kohei Hashimoto, Wakano Ogawa, Toshihiro Nishioka, Tomofusa Tsuchiya, Teruo Kuroda

**Affiliations:** Department of Molecular Microbiology, Graduate School of Medicine, Dentistry, and Pharmaceutical Sciences, Okayama University, Tsushima, Okayama, Japan; University of Birmingham, United Kingdom

## Abstract

Multidrug efflux pumps play an important role as a self-defense system in bacteria. Bacterial multidrug efflux pumps are classified into five families based on structure and coupling energy: resistance−nodulation−cell division (RND), small multidrug resistance (SMR), major facilitator (MF), ATP binding cassette (ABC), and multidrug and toxic compounds extrusion (MATE). We cloned a gene encoding a MATE-type multidrug efflux pump from *Streptococcus pneumoniae* R6, and designated it *pdrM*. PdrM showed sequence similarity with NorM from *Vibrio parahaemolyticus*, YdhE from *Escherichia coli,* and other bacterial MATE-type multidrug efflux pumps. Heterologous expression of PdrM let to elevated resistance to several antibacterial agents, norfloxacin, acriflavine, and 4′,6-diamidino-2-phenylindole (DAPI) in *E. coli* KAM32 cells. PdrM effluxes acriflavine and DAPI in a Na^+^- or Li^+^-dependent manner. Moreover, Na^+^ efflux via PdrM was observed when acriflavine was added to Na^+^-loaded cells expressing *pdrM*. Therefore, we conclude that PdrM is a Na^+^/drug antiporter in *S. pneumoniae*. In addition to *pdrM*, we found another two genes, spr1756 and spr1877,that met the criteria of MATE-type by searching the *S. pneumoniae* genome database. However, cloned spr1756 and spr1877 did not elevate the MIC of any of the investigated drugs. mRNA expression of spr1756, spr1877, and *pdrM* was detected in *S. pneumoniae* R6 under laboratory growth conditions. Therefore, spr1756 and spr1877 are supposed to play physiological roles in this growth condition, but they may be unrelated to drug resistance.

## Introduction

*Streptococcus pneumoniae* is the most common pathogenic bacterium in community-acquired pneumonia [1], [2], [3], [4], [5]. In Japan, it has been reported that *S. pneumoniae* is detected in 10–36% of investigated episodes of community-acquired pneumonia [6], [7], [8], [9]. This bacterium colonizes the upper respiratory tract and sometimes causes pneumonia, but it is also responsible for acute otitis media, bacteremia, and bacterial meningitis [10]. β-lactams and macrolides are usually used to treat *S. pneumoniae* infections, but the recent increase in the prevalence of antibiotic-resistant *S. pneumoniae* is a serious problem in clinical sites worldwide [8], [11], [12], [13], [14], [15], [16].

The frequency of isolating penicillin-resistant *S. pneumoniae* (PRSP), including penicillin-intermediate resistant *S. pneumoniae* (PISP), is as high as 25–50% in Japan [17], [18]. This, in addition to emerging multidrug-resistant *S. pneumoniae* (MDRSP), makes therapy difficult.

Several mechanisms contribute to bacterial resistance to drugs: 1) inactivation of drugs by degradation or modification, 2) modification of drug targets, 3) emergence of a bypass mechanism that is not inhibited by drugs, 4) changes in membrane permeability for drugs, and 5) drug efflux from cells. Among these five mechanisms, drug efflux, especially multidrug efflux, is known to play an important role in multidrug resistance in bacteria [19], [20], [21], [22].

Multidrug efflux pumps actively expel various chemical compounds, including antibiotics, dyes, and detergents. So far, three drug efflux pumps, PmrA [23], MefE [24], and PatAB [25] have been reported in *S. pneumoniae*. PmrA and MefE are classified as MF-type pumps and PatAB is an ABC-type efflux pump [26], [27]. However, more drug efflux pump genes are predicted to exist in the *S. pneumoniae* genome and remain uncharacterized (http://www.streppneumoniae.com/).

We found a new gene for multidrug resistance in *S. pneumoniae* by shot-gun cloning. The gene was predicted to encode a MATE-type efflux pump because of the similarity of its primary structure with known MATE-type efflux pumps [28].

The crystal structure of the MATE-type efflux pump, NorM from *Vibrio cholerae* was solved in 2010 and results showed that this protein possesses twelve transmembrane helices, composed of two bundles of six transmembrane helices (TMs 1–6 and TMs 7–12) [29]. Coupling with Na^+^ and substrate is an interesting characteristic of MATE-type efflux pumps, and this phenomenon has been reported in most MATE-type efflux pumps (*e.g.* NorM from *V. parahaemolyticus* [30], VcmA from *V. cholerae* non-O1 [31], VmrA from *V. parahaemolyticus* [32], VcrM from *V. cholerae* non-O1 [33], HmrM from *Haemophilus influenzae* Rd [34], and NorM from *Neisseria gonorrhoeae* [35]). Here we report the characteristics of a novel MATE-type efflux pump from *S. pneumoniae*, including its Na^+^ coupling ability.

## Methods

### Bacterial Strains and Growth

*S. pneumoniae* R6 (ATCC number, BAA-255), which was purchased from the American Type Culture Collection, was used as a source of chromosomal DNA. *S. pneumoniae* was grown in brain heart infusion (BHI, Difco) at 37°C for 16 hr∼24 hr without shaking. *E. coli* KAM32 (Δ*acrB*, Δ*ydhE*) [32], a drug-hypersusceptible strain, was used as a cloning host. *E. coli* was grown aerobically in LB broth at 37°C.

### Cloning of the *pdrM* Gene

Chromosomal DNA was prepared from *S. pneumoniae* R6 by the method of Berns and Thomas [36]. Chromosomal DNA was partially digested with *Sau*3AI, and fragments ranging in size from 3 to 10 kbp were separated by sucrose density gradient centrifugation. Plasmid pHY300PLK (Takara Co.) was digested with *Bam*HI, dephosphorylated with shrimp alkaline phosphatase (Roche Diagnostics K.K.), and then ligated to chromosomal DNA fragments with a Ligation-Convenience Kit (Nippon Gene Co.). Competent *E. coli* KAM32 cells were transformed with recombinant plasmids and incubated at 37°C for 48 h on LB plates containing an antimicrobial agent. We also used 10 µg/ml erythromycin and 10 µg/ml tetraphenylphosphonium chloride to clone drug efflux pump genes, but could not isolate any candidates. We isolated eight candidates from a plate containing 5 µg/ml acriflavine. Single colonies were isolated on the same medium as the selection medium, and plasmids were extracted from cells. *E. coli* KAM32 was re-transformed with the plasmids to confirm that it was responsible for the observed resistance to the antimicrobial agent used for the selection. Subsequently, restriction maps of candidate plasmids were determined.

### PCR Cloning of Putative MATE Efflux Pump Genes

The *pdrM* gene was also cloned by PCR to compare its activity with the products of spr1756 and spr1877 under the same promoter. A DNA fragment containing the *pdrM*, spr1756, or spr1877 gene was amplified by PCR using *S. pneumoniae* R6 chromosomal DNA as a template. Primers used for gene cloning are listed in Table S1. The amplified DNA fragment containing each gene was ligated into *Bam*HI sites of pSTV29 (Takara Co.) or pUC19.

### Drug Susceptibility Test

The MICs of various antimicrobial agents were determined by the microdilution method according to the recommendations of the Japanese Society of Chemotherapy (Japanese Society of Chemotherapy, 1990). Briefly, MICs were determined in Mueller–Hinton broth (Difco) containing each compound in a twofold serial dilution series. About 10^4^ cells were inoculated in each well of a microtiter plate, incubated in the test medium at 37°C for 24 h, and growth was examined visually. The MIC of each compound was defined as the lowest concentration that prevented visible growth.

### Energy Starvation and Loading Fluorescent Substance

*E. coli* KAM32 harboring the recombinant plasmid was grown in LB medium at 37°C until OD_650_≈0.8. Cells were harvested and washed twice with modified Tanaka buffer (pH 7.0) containing 2 mM MgSO_4_ [37]. Cells were resuspended in this buffer containing acriflavine (final concentration: 4.2 µM) or 4', 6-diamidino-2-phenylindole (DAPI, final concentration: 5 µM). Then, in order to load fluorescent dye into the cells, cells were incubated at 37°C for 2.5 hr in the presence of 100 µM carbonylcyanide-*m*-chlorophenylhydrazone (CCCP). Control cells and *pdrM*-possessing cells were normalized according to cell density and fluorescent intensity. By initially adjusting the cell density of control and sample cells, the fluorescent intensity was roughly adjusted. Then the fluorescent intensity was slightly adjusted last before the assay.

### Efflux of Fluorescent Substance

Efflux of acriflavine or DAPI was carried out as previously described [32], [38]. Energy starved and fluorescent dye -loaded cells were washed three times with 0.1 M 3-morpholinopropanesulfonic acid (MOPS)- tetramethylammonium hydroxide (TMAH) (pH 7.0) containing 2 mM MgSO_4_ and the appropriate concentration of fluorescent dye (4.2 µM acriflavine or 5 µM DAPI), and were resuspended in the same buffer. Fluorometric measurements were performed at 37°C with a Hitachi F-2000 fluorescence spectrometer. Excitation and emission wavelengths used for the acriflavine efflux assay were 468 nm and 499 nm, respectively, whilst 332 nm and 462 nm were used for the DAPI efflux assay. After preincubation at 37°C for 4 min, lactate-TMAH (pH 7.0) (final concentration 20 mM) was added to energize the cells, and then NaCl, LiCl, or KCl (final concentration 10 mM) was added to the assay mixture.

### Acriflavine Efflux from Cells Induced by an Artificial Inward Na^+^ Gradient

Based on our previous report, accelerated acriflavine efflux was measured by generating a transient Na^+^ gradient [30]. Energy-starved and fluorescent dye -loaded cells were washed three times with 0.1 M MOPS-TMAH (pH 7.0) containing 2 mM MgSO_4_ and 4.2 µM acriflavine, and were resuspended in the same buffer. Buffer containing NaCl or KCl (final concentration 100 mM) was incubated at 37°C for 4 min to draw a base line. Then, prepared cells were added to the incubating buffer to make cells impose an inward Na^+^ gradient and acriflavine fluorescence at 499 nm was monitored with excitation at 468 nm.

### Efflux of Na^+^ Induced by Addition of Acriflavine

Na^+^ efflux by the addition of acriflavine was observed as previously described [30]. Briefly, *E. coli* KAM32 harboring recombinant plasmids were grown in modified Tanaka medium [37] supplemented with 1% tryptone, 5 mM MgSO_4_, and 10 mM melibiose at 30°C [39]. Cells were harvested at the late-exponential phase of growth, and washed three times with 0.1 M MOPS-TMAH (pH 7.0) containing 5 mM MgSO_4_, and were resuspended in the same buffer. A portion of this suspension was diluted to approximately 9 mg protein/ml in the same buffer containing 250 µM NaCl.

Cells were incubated at 25°C in a plastic vessel with rapid stirring, and N_2_ gas was blown continuously to maintain anaerobic conditions. A Na^+^-electrode (Radiometer, Copenhagen, Denmark) and reference electrode were put into the vessel [40]. Methyl-β-D-galactopyranoside (Mβgal), a substrate of the melibiose transporter, which is coupled to Na^+^ efflux [41], was added (final concentration of 5 mM) to induce Na^+^ uptake into cells. Thereafter, acriflavine was added to the assay mixture (final concentration, 200 µM). Changes in the Na^+^ concentration of the assay medium were monitored with a Na^+^ electrode. Calibration was carried out by the addition of known amounts of NaCl.

### Reverse Transcriptase-Polymerase Chain Reaction (RT-PCR) Analysis

Total RNA was extracted using the QIAGEN RNeasy Mini Kit (QIAGEN Inc.) from *S. pneumoniae* R6 cultures grown in BHI broth until the exponential phase. For efficient RNA extraction, cells were broken well with a QIA shredder (QIAGEN Inc.) prior to RNA extraction. The extracted total RNA was applied to RT-PCR with the QIAGEN One-Step RT-PCR Kit (QIAGEN Inc.). Primers used for RT-PCR are listed in Table S1. PCR without the reverse-transcriptase reaction was performed to confirm no detectable DNA contamination. RT-PCR products were analyzed by 3% Agarose X (Nippon Gene Co.) gel electrophoresis.

### Gene Disruption in *Bacillus subtilis*

*Bacillus subtilis* 168, RIK356, RIK482, and RIK483 were kind gifts from Prof. Kawamura in Rikkyo University, College of Science. *B. subtilis* strains were cultured aerobically in LB broth at 37°C. A GMM agar plate (0.6% KH_2_PO_4_, 1.4% K_2_HPO_4_, 0.2% (NH_4_)_2_SO_4_, 0.1% Na citrate 2 H_2_O, 0.5% glucose, 0.02% MgSO_4_, 1.5% agar) was used to select a strain using the auxotrophic phenotype [42]. The following concentrations of antibiotics were added when needed: kanamycin (final concentration 5 µg/ml), erythromycin (final concentration 0.5 µg/ml), chloramphenicol (final concentration 10 µg/ml), and tetracycline (final concentration 10 µg/ml). Gene disruption on *B. subtilis* was performed according to the method by Ochi et al [43]. Primers used for gene disruption were shown in Table S1.

## Results

### Functional Cloning of *pdrM*

We cloned genes relating to drug resistance from *S. pneumoniae* R6 [44] using a shot-gun method, and isolated eight hybrid plasmids that conferred acriflavine resistance to the heterologous host, *E. coli* KAM32. *E. coli* cells possessing hybrid plasmids showed resistance not only to acriflavine but also to norfloxacin, suggesting that the cloned gene is related to multidrug resistance. All hybrid plasmids possessed a common region of approximately 3 kbp, and we chose one of the eight plasmids, pHAP5, for the following analysis (Figure S1).

### Sequence Analysis of pHAP5

We identified the DNA region cloned in pHAP5 by comparing the partially determined sequence of pHAP5 with the *S. pneumoniae* R6 genome sequence (http://www.streppneumoniae.com/). The cloned region on pHAP5 contained one intact ORF (spr1052) and parts of two additional ORFs (spr1051 and spr1053).

spr1053 was deduced to encode dihydroorotase [45], an enzyme in the ribonucleotide biosynthetic pathway, whilst spr1051 was deduced to encode an AcoA homolog. AcoA was reported to be an alpha chain of thiamine-PPi dependent acetoin dehydrogenase in *B. subtilis* [46].

spr1052 encodes a protein similar to NorM from *V. parahaemolyticus* (ACCESSION No. AB010463.1), YdhE from *E. coli* (ACCESSION No. U00096.2), and AbeM from *Acinetobacter baumannii* (ACCESSION No. AB204810.2). These proteins belong to the MATE-type multidrug efflux pump (Table 1, Figure S2), leading us to predict that spr1052 also encodes a MATE-type multidrug efflux pump [28], [47]. We named this gene *pdrM* (Pneumococcal Drug Resistance).

**Table 1:** Similarity of putative MATE transporters in *S. pneumoniae* R6 with characterized MATE-type transporters.

	spr1052 (PdrM)	spr1756	spr1877
**Cluster 1** *			
**spr1756 (** ***Sp*** **)**	22	–	59
**spr1877 (** ***Sp*** **)**	63	59	–
**AbeM (** ***Ab*** **)**	72	60	20
**NorM (** ***Vp*** **)**	71	60	49
**HmrM (** ***Hi*** **)**	69	18	63
**VcmA (** ***Vc*** **)**	69	35	31
**Cluster 2** *			
**ALF5 (** ***At*** **)**	62	58	10
**ERC1 (** ***Sc*** **)**	55	48	23
**hMATE1 (** ***Hs*** **)**	20	49	17
**TT12 (** ***At*** **)**	24	41	22
**Cluster 3** *			
**DinF (** ***Ec*** **)**	32	59	29
**VmrA (** ***Vp*** **)**	28	63	41
**MepA (** ***Sa*** **)**	53	43	62

* The classification was referred to the result of analysis with ClustalW and reference [47].

Genetyx ver. 7.0.8 was used to estimate the scores of identity and similarity.

*Sp : Streptococcus pneumoniae, Ab : Acinetobacter baumannii, Hi : Haemophilus influenzae, Vc : Vibrio cholerae, At : Arabidopsis thaliana, Sc : Saccharomyces cerevisiae, Hs : Homo sapiens, Ec : Escherichia coli, Vp : Vibrio parahaemolyticus, Sa : Staphylococcus aureus.*

The *pdrM* ORF was estimated to be 1,359 bp and was presumed to encode a hydrophobic protein of 453 amino acid residues. A terminator-like sequence was found downstream of spr1053 (upstream of *pdrM*) and a promoter-like sequence was found upstream of *pdrM* (Software: Genetyx Version 7.0.8). No promoter or promoter-like sequence was identified around the *Bam*HI cloning site on the pHY300PLK cloning vector. Thus, spr1053 and *pdrM* do not appear to be operonic and *pdrM* cloned on pHY300PLK is presumed to be expressed from its own promoter in *E. coli* cells. A terminator-like sequence was found downstream of *pdrM*, and a promoter-like sequence was found upstream of spr1051. For these reasons, *pdrM* appears to be a monocistronic gene.

### Drug Susceptibility Testing

The MIC of various antibiotics was measured in *E. coli* KAM32 transformed with the *pdrM*-expressing plasmid (Table 2). The MIC of acriflavine in KAM32/pHAP5 was four times higher than that of KAM32/pHY300PLK. The MICs of norfloxacin and DAPI in KAM32/pHAP5 also increased four and sixteen times, respectively, demonstrating that *pdrM* is indeed a gene involved in multidrug resistance.

**Table 2:** Drug susceptibility in cells transformed with *pdrM.*

MIC (µg/ml)						
	*E. coli* KAM32		*B. subtilis* TN26			*B. subtilis* 168
Antimicrobial agents	pHY300PLK (control)	pHAP5 (*pdrM*)		pHY300PLK (control)	pHAP5 (*pdrM*)	(parental strain)
** **Norfloxacin	0.015	0.06		0.25	0.25	1
** **Ciprofloxacin	0.004	0.008		0.25	0.125	ND
** **Enoxacin	0.06	0.06		0.5	0.5	ND
** **Ofloxacin	0.015	0.015		ND	ND	ND
** **Chloramphenicol	0.25	0.5		ND	ND	4
** **Erythromycin	2	4		ND	ND	0.06
** **Acriflavine	2	8		0.25	0.25	1
** **DAPI	0.125	2		0.5	0.5	ND
** **EtBr	4	4		0.5	0.5	4
** **Benzalkonium Cl	4	4		ND	ND	2
** **Rhodamine 6G	8	8		ND	ND	1
** **TPPCl	4	4		4	4	64

DAPI: 4′,6-diamidino-2-phenylindole, EtBr: ethidium bromide,

TPPCl: tetraphenylphosphonium chloride, ND: not determined.

We suspected that PdrM may be more active in Gram positive bacteria than in *E. coli* because *S. pneumoniae* is a Gram positive bacterium. *B. subtilis* is one of the legally recognized hosts of genetic transformation in Japan, and we planned to investigate the activity of PdrM in *B. subtilis*. However, we knew that the basic drug resistant levels of wild type strains often prevented detection of the activity of a drug efflux pump, and we constructed *B. subtilis* TN26 from *B. subtilis* 168 before the investigations. Six genes (*bmr* [48], *ebrAB* [49], *blt* [50], *bmrA* [51], *yerP* [52], and *bmr3* [53]) for the drug efflux pump were disrupted in *B. subtilis* TN26. The MIC of tetraphenylphosphonium chloride in *B. subtilis* TN26 was sixteen times lower than that of the parental strain 168 and the MICs of norfloxacin, acriflavine, and ethidium bromide were also four times lower than that of the parental strain (Table 2). We then measured the drug resistant level in *B. subtilis* TN26 transformed with *pdrM*. However, *pdrM* was unable to elevate the MIC of the chemicals we investigated although the host was the same Gram positive bacterium *B. subtilis* as *S. pneumoniae*. We considered that the slightly decreased MIC of ciprofloxacin were in a range for error. We confirmed the mRNA expression of *pdrM* in *B. subtilis* TN26/pHAP5 using RT-PCR (Figure S3), and there may be some difficulty with translation or transition to the membrane in *B. subtilis*. As another reason, it may be possible that the basic resistant levels for quinolones and DAPI are still too high in *B. subtilis* TN26 to detect MIC changes.

### Drug Efflux Assay

**Figure 1:**
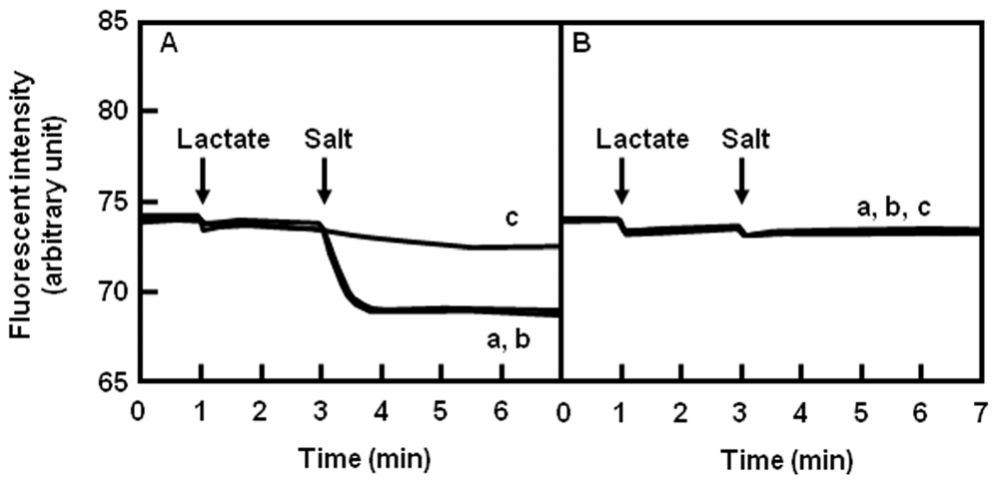
Effect of monovalent cations on energy-dependent DAPI efflux mediated by PdrM. Lactate-TMAH (final concentration 20 mM, pH 7.0) was added to the assay mixture (at the first arrow). At the time point indicated by the second arrow, either NaCl (curve a), LiCl (curve b), or KCl (curve c) was added to the assay mixture (final concentration 10 mM). (A) *E. coli* KAM32/pHAP5, (B) *E. coli* KAM32/pHY300PLK (control). The fluorescence of free DAPI and DAPI binding with DNA was detected in the DAPI assay. However, the fluorescence from DAPI binding with DNA was stronger than free DAPI and the fluorescent intensity decreased when DAPI was pumped out of the cells.

PdrM recognized DAPI and acriflavine as its good substrates. Therefore, we investigated the efflux activity of DAPI and acriflavine in *E. coli* KAM32/pHAP5 (Figure 1). No active efflux activity was observed when only lactate was added to the cell suspension. Consequently, NaCl was added to the assay mixture, and significant DAPI efflux was observed. The addition of LiCl also activated DAPI efflux as strongly as NaCl, although the efflux of DAPI was not observed when KCl was added to the assay mixture. Thus, Na^+^ and Li^+^ cations, but not Cl^-^, facilitate the efflux activity of PdrM. A similar result was observed when acriflavine was used as the substrate for the efflux assay (data not shown).

**Figure 2:**
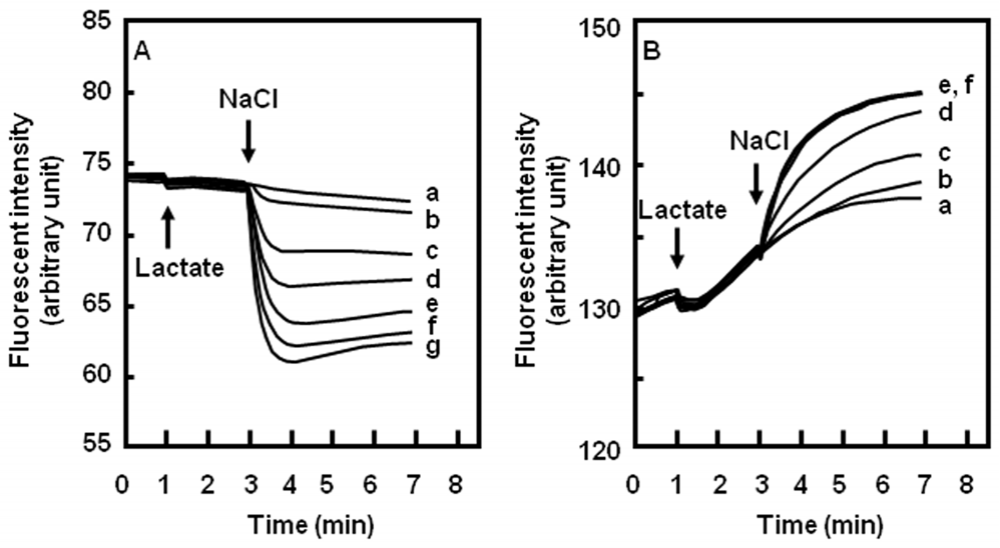
Salt concentration-dependent fluorescent dye efflux via PdrM. DAPI efflux activity (A). The concentration of NaCl or LiCl is (a) 0 mM, (b) 1 mM, (c) 10 mM, (d) 30 mM, (e) 50 mM, (f) 80 mM, and (g) 100 mM. Acriflavine efflux activity (B). The concentration of NaCl or LiCl is (a) 0 µM, (b) 10 µM, (c) 100 µM, (d) 1 mM, (e) 10 mM, and (f) 20 mM. Acriflavine fluorescence was quenched in cells because of its concentration. When an energy source was added, acriflavine was expelled and acriflavine fluorescence increased. Different from DAPI, the fluorescence of acriflavine does not change by DNA binding.

Next, we investigated the dependency of DAPI efflux on NaCl concentrations (Figure 2(A)). DAPI efflux in *E. coli* KAM32/pHAP5 was accelerated with increasing NaCl concentrations, and reached a plateau at 100 mM NaCl. DAPI efflux was also stimulated in a concentration-dependent manner by LiCl (data not shown). DAPI efflux curves almost coincided when the same concentration of LiCl was added (data not shown). Thus, NaCl and LiCl are compatible with one another in DAPI efflux.

It was also observed that acriflavine was effluxed in a Na^+^ concentration-dependent manner (Figure 2(B)). Incongruously, however, DAPI efflux was maximal at ∼100 mM Na^+^ whereas maximal acriflavine efflux occurred at ∼10 mM Na^+^. Various reasons were conceived to explain this discrepancy. However, the most possible reason we could think of was that the lowest concentrations of DAPI and acriflavine detectable by our luminometric method may be different. Alternatively, stoichiometry with Na^+^ may be different between DAPI and acriflavine.

### Na^+^ Efflux Elicited by Acriflavine Influx

**Figure 3:**
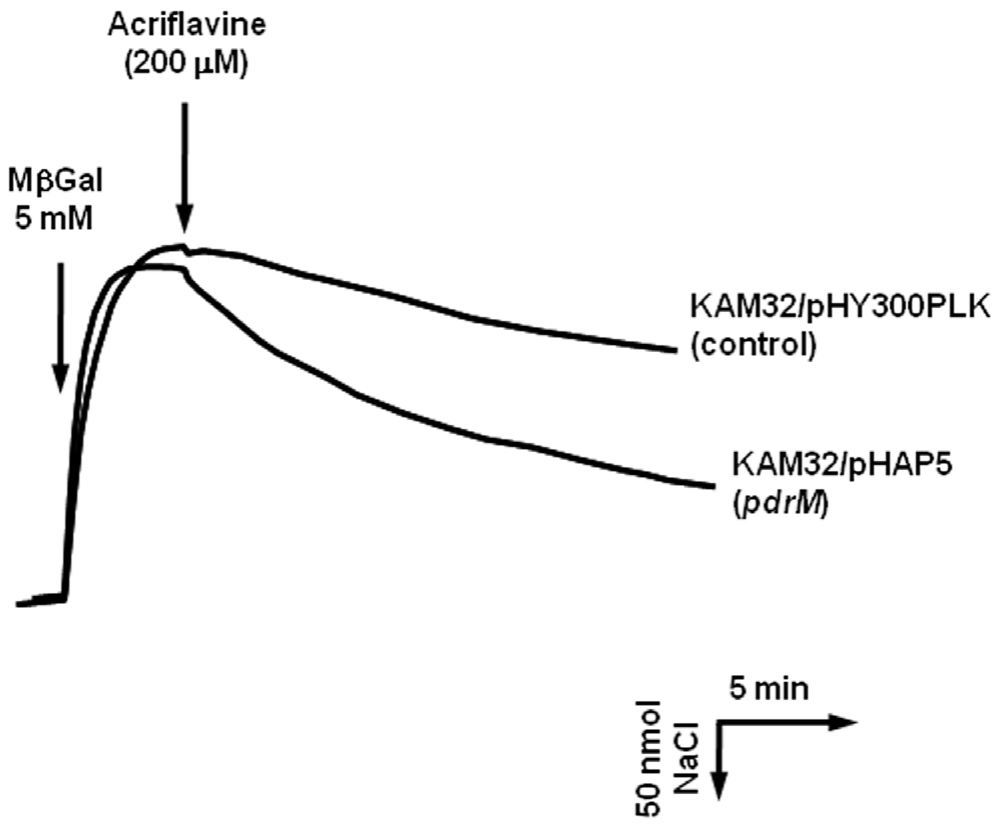
Na^+^ efflux from Na^+^-loaded cells elicited by an inwardly directed acriflavine gradient. Cells of *E. coli* KAM32/pHAP5 or *E. coli* KAM32/pHY300PLK (control) were diluted with 0.1 M MOPS-TMAH (pH7.0) until approximately 9 mg protein/ml. Na^+^ was loaded to cells via MelB by the addition of Mβgal (the first arrow), and acriflavine (final 200 µM) was added to the assay mixture at the time point indicated by the second arrow. Upward deflection of the curve indicates Na^+^ influx into cells and downward deflection indicates Na^+^ efflux from cells.

Several reported MATE-type multidrug efflux pumps are known to utilize Na^+^ to extrude their substrates [30], [31], [32], [33], [34], [35]. Therefore, we investigated whether Na^+^ movement accompanied substrate extrusion via PdrM (Figure 3). Indeed, Na^+^ was extruded when acriflavine was added to a cell suspension of *E. coli* expressing *pdrM*. This result demonstrates that PdrM acts as an antiporter to exchange Na^+^ and acriflavine, and that Na^+^ is utilized as a coupling cation to transport drugs.

**Figure 4:**
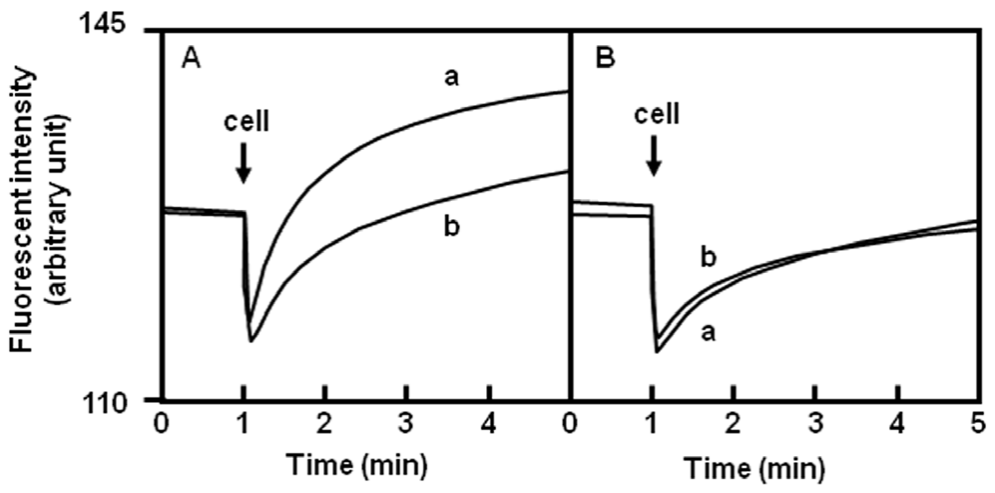
Acriflavine efflux from cells elicited by an inwardly directed artificial Na^+^ gradient. An inwardly directed chemical gradient of Na^+^ was imposed by the addition of the cell suspension into the assay medium containing salt at the time point indicated by the arrow. (A) *E. coli* KAM32/pHAP5, (B) *E. coli* KAM32/pHY300PLK. The final concentration of the salt was 100 mM NaCl (curve a) or 100 mM KCl (curve b). The assay was performed at 37°C.

Acriflavine should be expelled in the presence of an artificial Na^+^ concentration gradient if PdrM antiports Na^+^ and acriflavine. To investigate this, acriflavine efflux via PdrM was measured in cells with a reduced intracellular Na^+^ concentration. Energy-starved and acriflavine-loaded cells, in which Na^+^ concentrations are low, were prepared. Then, the cells were rapidly added to the assay mixture containing NaCl (final concentration 100 mM) to form an inward Na^+^ gradient. As a result, acriflavine was effluxed from *pdrM*-possessing cells in response to the Na^+^ gradient (Figure 4).

In contrast, this phenomenon was not observed with KCl instead of NaCl. This result also shows that acriflavine efflux is dependent on Na^+^ and is not due to the effect of osmotic pressure or Cl^−^.

**Figure 5:**
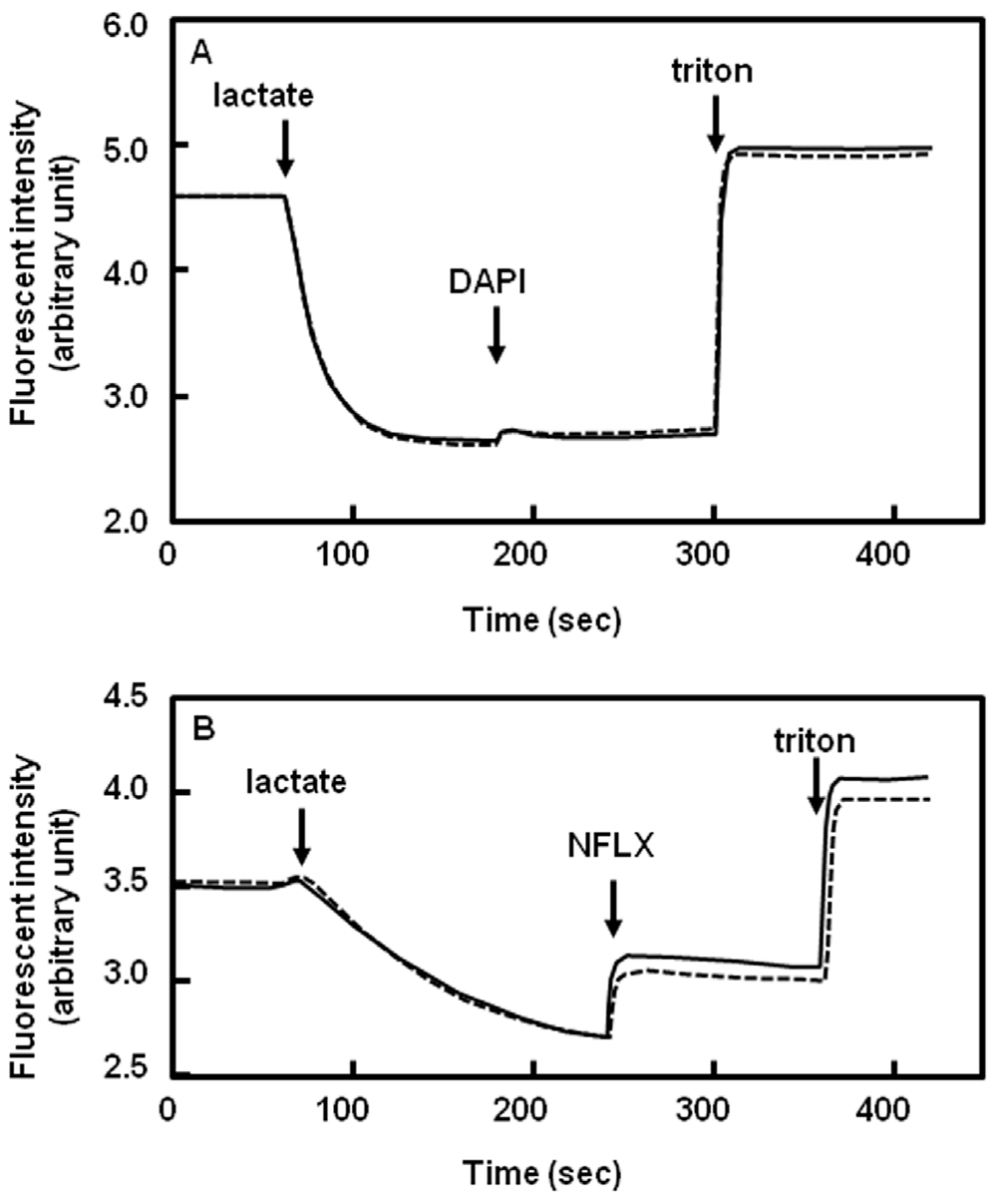
H^+^ efflux assay in the inverted vesicle. H^+^ efflux activity was tested with inverted membrane vesicles from *E. coli* KAM32/pHAP5 (solid line) and KAM32/pHY300PLK (dashed line). Each reagent was added at the time point shown by the arrow. The final concentration of lactate, potassium salt was 5 mM. Potassium lactate adjusted at pH 7.0 was used for the assay. The final concentrations of DAPI and NFLX were 5 µM and 250 µM, respectively, and the final concentration of triton X100 was 0.0125%. The assay mixture is 0.1 M Tricine-KOH (pH 8.0) containing 5 mM MgSO_4_ (panel A). 0.1 M MOPS-KOH containing 5 mM MgSO_4_ was used for the assay at pH 6.5 (Panel B) and pH 7.0 (data not shown). The result at pH 7.0 was similar to panel A. An assay with NFLX at pH 8.0 was also performed, but H^+^ efflux activity was not observed.

We also investigated the antiport activity of norfloxacin and H^+^ via PdrM using a quinacrine fluorescence method [54], but the movement of H^+^ was not induced by the addition of norfloxacin or DAPI (Figure 5).

### Putative MATE Efflux Pumps in *S. pneumonaie* R6

**Figure 6:**
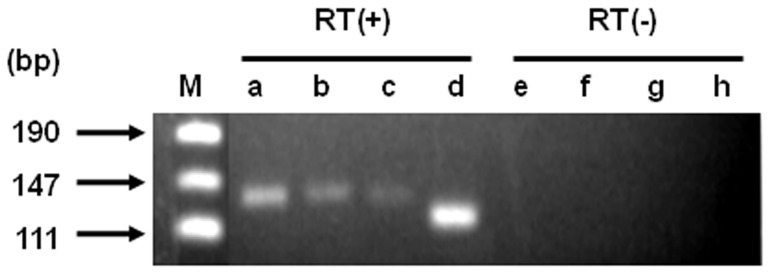
RT-PCR analysis in *S. pneumoniae* R6. One ng total RNA was used for the template for one reaction of RT-PCR, and the reaction was repeated for 24 cycles. pUC19 digested with *Msp* I was used as a size marker (lane M). *pdrM* (lane a, e), spr1756 (lane b, f), spr1877 (lane c, g). The expression of the *atpB* was used as an internal control (lane d, h). Reverse transcriptional reactions were submitted on samples in lane a, b, c, and d, and were not on samples in lane e, f, g, and h.

We found three genes – spr1756, spr1877, and *pdrM* – that met the criteria of the MATE family by searching the *S. pneumoniae* genome database (Figure S2). Of these three genes, spr1756 was reported to encode a protein whose function is similar to DinF [55]. Recently, Tocci et al reported three ORFs (SP1166, SP1939, and SP2065) belonging to MATE in *S. pneumoniae* DP1004 [56]. From the putative amino acid sequence, SP1166, SP1939, and SP2065 in *S. pneumoniae* DP1004 are thought to encode the orthologs of *pdrM*, spr1756, and spr1877 in *S. pneumoniae* R6, respectively. We cloned spr1756, spr1877, and *pdrM* by PCR, and measured the MIC of several antibiotics in *E. coli* KAM32 expressing the cloned gene (Table 3). Expression of *pdrM* cloned by PCR also led to elevated resistance against acriflavine and DAPI to the same extent as the *pdrM* cloned functionally (described above). spr1756 and spr1877 did not elevate the MIC of any of the drugs. mRNA of all three genes, however, were detected in *S. pneumoniae* R6 under laboratory growth conditions (Figure 6). Tocci et al constructed a spr1756-disrupted mutant from *S. pneumoniae* R6 and revealed that the MICs of moxifloxacin and levofloxacin were slightly lower in the mutant. Spr1756 may be able to efflux moxifloxacin; however, we speculate that the main role of spr1756 may be different from expelling antimicrobial chemicals in *S. pneumoniae* R6. spr1877 is also expected to have a physiological role that is presumably unrelated to drug resistance.

**Table 3:** Drug susceptibility in cells transformed with genes predicted from genome information.

MIC (µg/ml)				
	host : *E. coli* KAM32			
Antimicrobial agents	pSTV29 (control)	*pdrM* (spr1052)	spr1756	spr1877
** **Oxacillin	1	2	2	2
** **Cloxacillin	2	2	4	4
** **Erythromycin	4	4	4	4
** **Norfloxacin	0.03	0.03	0.03	0.03
** **Acriflavine	2	16	2	2
** **Hoechst 33342	0.5	1	0.5	0.5
** **DAPI	0.25	2	0.25	0.25
** **Methyl viologen	128	128	128	128
** **Novobiocin	2	2	2	4
** **Trimethoprim	0.03	0.06	0.03	0.03
** **Safranin O	4	8	4	4

DAPI: 4′,6-diamidino-2-phenylindole.

MATE-type pumps are phylogenetically classified into three clusters. PdrM is categorized into cluster 1 [57], along with typical bacterial MATE-type efflux pumps such as NorM from *V. parahaemolyticus* and YdhE from *E. coli* (Figure S2). In contrast, spr1756 and spr1877 are classified into cluster 3 [57]. Bioinformatic analysis predicted that many bacterial membrane proteins belong to cluster 3, but only a few of them, (*e. g.* MepA from *S. aureus* [58], VmrA from *V. parahaemolyticus* [32], and DinF from *Ralstonia solanacearum* [59]) were actually shown to possess the drug efflux activity. Indeed, DinF from *E. coli* is a well-known protein that belongs to cluster 3 in the MATE family but has not been reported to contribute to drug resistance. Proteins in cluster 3 may be rarely involved in drug resistance in bacteria.

What are the roles of spr1756 and spr1877? They are expressed under laboratory conditions, and we suggested that they have some as of yet undetermined role under these culture conditions. Muñoz-Elías’s group isolated a mutant possessing a transposon in spr1756 (*dinF*), corresponding to sp1939 in *S. pneumoniae* TIGR4 [60], and the transposon mutant in sp1939 formed poorer biofilms than the parent, *S. pneumoniae* TIGR4. Their results may support our hypothesis that spr1756 and spr1877 gene products play some role unrelated to drug resistance in *S. pneumoniae,* although a specific physiological role of spr1756 is still unknown.

## Discussion

We cloned a MATE-type multidrug efflux pump gene, *pdrM* from *S. pneumoniae* R6. PdrM effluxes acriflavine and DAPI in a Na^+^ dependent manner. We could not detect the antiport activity of norfloxacin (or DAPI) and H^+^ via PdrM though we previously found AbeM from *A. baumannii*, which is a H^+^-coupled MATE-type efflux pump [61]. Therefore, we considered H^+^ was unlikely to be moved via PdrM.

Sodium circulation in *S. pneumoniae* has not been investigated extensively. Kakinuma et al reported that Na^+^-ATPase classified into V-type ATPase in *Enterococcus hirae* was closely related to Streptococci [62] and the Na^+^/proton antiporter in *Enterococcus faecalis* [63]. In *Streptococcus bovis*, sodium-dependent transport of neutral amino acids was reported in both whole cells and membrane vesicles [64]. Because these closely related bacteria possess systems coupling with Na^+^, we assumed that the existence of systems to utilize Na^+^ should also be expected in *S. pneumoniae.* We searched the genome database of *S. pneumoniae* R6 to determine whether the genes encoding similar proteins to the Na^+^-ATPase, NtpFIKECGABDHJ from *E. hirae* or various known Na^+^/proton antiporters (*e.g.* NhaP from *Pseudomonas aeruginosa* [65], NhaB from *E. coli* [66], MnhA from *S. aureus* [67], and human Na^+^/H^+^ antiporter, NHE-2 [68]) are present or not. However, no such genes were found in the genome in *S. pneumoniae* R6.

We did find spr0573, described to be a sodium/hydrogen exchanger family protein Nha2, in the genome database (http://www.ncbi.nlm.nih.gov/nuccore/NC_003098), but spr0573 is barely similar to NhaP from *P. aeruginosa* (less than 40% similarity). spr0573 also showed little similarity with NhaB from *E. coli*, MnhA from *S. aureus*, human NHE-2, and other known Na^+^/H^+^ antiporters. Therefore, it is doubtful whether Nha2 (spr0573) is actually a Na^+^/H^+^ antiporter.

We found at least three proteins possibly using Na^+^ as a coupling ion based on the genome information of *S. pneumoniae* R6. They are spr1598, spr0369, and spr0648. Among them, we could not find any reports relating to spr0648. spr1598 is presumed to encode a serine transporter because of its high similarity (90%) to SstT from *E. coli* [69]. spr0369 has been renamed *dagA* and it has been proposed to be a protein in the sodium/alanine symporter family. More than half of N-terminal of DagA from *S. pneumoniae* R6 is highly similar to DagA from the marine bacterium, *Alteromonas haloplankti*s [70].

The presence of these genes and *pdrM* suggests that *S. pneumoniae* also utilizes secondary transporters coupled with Na^+^. Therefore, we think that *S. pneumoniae* possesses machineries to form a Na^+^ motive force although we cannot predict any possible candidate genes now.

Na^+^ and Li^+^ significantly promoted expulsion of the fluorescent dyes, acriflavine and DAPI via PdrM, whilst K^+^ did not. Minimal efflux of the fluorescent dyes was observed in both KAM32/pHAP5 and control cells when KCl was added to the assay mixture, and this phenomenon was observed in NorM from *V. parahaemolyticus* [30]. This result suggested that *E. coli* KAM32 possesses a weak acriflavine efflux system that is promoted in the presence of KCl, and is independent of PdrM.

On the other hand, KAM32/pHAP5 seemed to show higher acriflavine efflux activity in Figure 4(A) than in Figure 4(B). This may have been caused by PdrM though acriflavine efflux was not observed in Figure 1 and 2. Contaminated Na^+^ in high concentration KCl solution may stimulate the activity of PdrM or K^+^ at high concentrations may play a role similar to Na^+^.

In summary, *pdrM* from *S. pneumoniae* has been revealed to be a MATE-type multidrug efflux pump. We would emphasize that PdrM is the first multidrug efflux pump that shows Na^+^-coupling availability in Gram-positive bacteria.

## Supporting Information

Figure S1 **Restriction map of plasmid pHAP5.** Horizontal bar indicates DNA regions derived from chromosomal DNA of *S. pneumoniae* R6. Arrows indicate ORFs of *pdrM* (spr1052), spr1051, and spr1053. The vector plasmids for pHAP5 and pUAP19 are pHY300PLK and pUC19, respectively.
(TIF)

Figure S2 **Phylogeny of MATE-type proteins in S. pneumoniae and related proteins.** MATE-type proteins were aligned in CLUSTAL W (Thompson JD, Higgins DG, Gibson TJ.(1994)Nucleic Acids Res. 22(22):4673–4680.) Accession number of each protein : NP_388962(YisQ (*B. subtilis*)), EHJ39019(MatE (*Clostridium difficile*)), AAF94694(NorM (*Vibrio cholerae* O1), YP_213507(BF3926 (*Bacteroides fragilis*), P45272 (HmrM (*Haemophilus influenzae*), AAC17857 (YojI (*B. subtilis*), BAB79260 (VcmA (*V. cholerae* non-O1), BAB68204 (VmrA (*V. parahaemolyticus*), AAH35288(SLC47A2 (*Homo sapiens*)), AEE79863 (TT12 (*Alabidopsis thaliana*)), NP_178499 (*A. thaliana*), AAH50592 (SLC47A1 (*Homo sapiens*)), NP_588077 (*Schizosaccharomyces pombe*), BAA31456(NorM (*V. parahaemolyticus*)), ZP_07363019(MepA (*Stapyulococcus aureus*)) AEE76777(ALF5 (*A. thaliana*)), DAA06723(ERC1 (*Saccharomyces cerevisiae*), BAD89844 (AbeM (*A. baumannii*)), CAK08901(RL3413 (*Rhizobium leguminosarum*)), YP_492187(DinF (*E. coli*)), CAE00499 (CdeA (*C. difficile*)), BAB79260 (VcmA (*V. cholerae* non-O1)), BAD89844 (AbeM (*Acinetobacter baumannii*), BAD98612 (VcmD (*V. cholerae* non-O1)), BAB70470(VcrM (*V. cholerae* non-O1)).
(TIF)

Figure S3 **mRNA expression of** ***pdrM*** **in** ***B. subtilis*** **TN26/pHAP5.** Panel A: RNA from *B. subtilis* TN26/pHAP5, Panel B: RNA from *B. subtilis* TN26/pHY300PLK Total RNA was extracted using the QIAGEN RNeasy Mini Kit (QIAGEN Inc.) from *B. subtilis* cultures grown in LB broth with 20 µg/ml tetracycline until the exponential phase. Genome-derived *atpB* mRNA expression in *B. subtilis* TN26 was used as a standard. Primers, atpB(B subtilis)F and atpB(B subtilis)R for the standard were shown in Table S1. Samples in RT(-) lanes were amplified without a reverse transcriptional reaction.
(TIF)

Table S1 **Primers used in this study.**
(DOCX)
