# Supplementary figures and images for: Functionally Cloned pdrM from Streptococcus pneumoniae Encodes a Na+ Coupled Multidrug Efflux Pump
Source: PLoS One. 2013 Mar 26;8(3):e59525. doi: 10.1371/journal.pone.0059525 (PMC3608713; doi:10.1371/journal.pone.0059525)

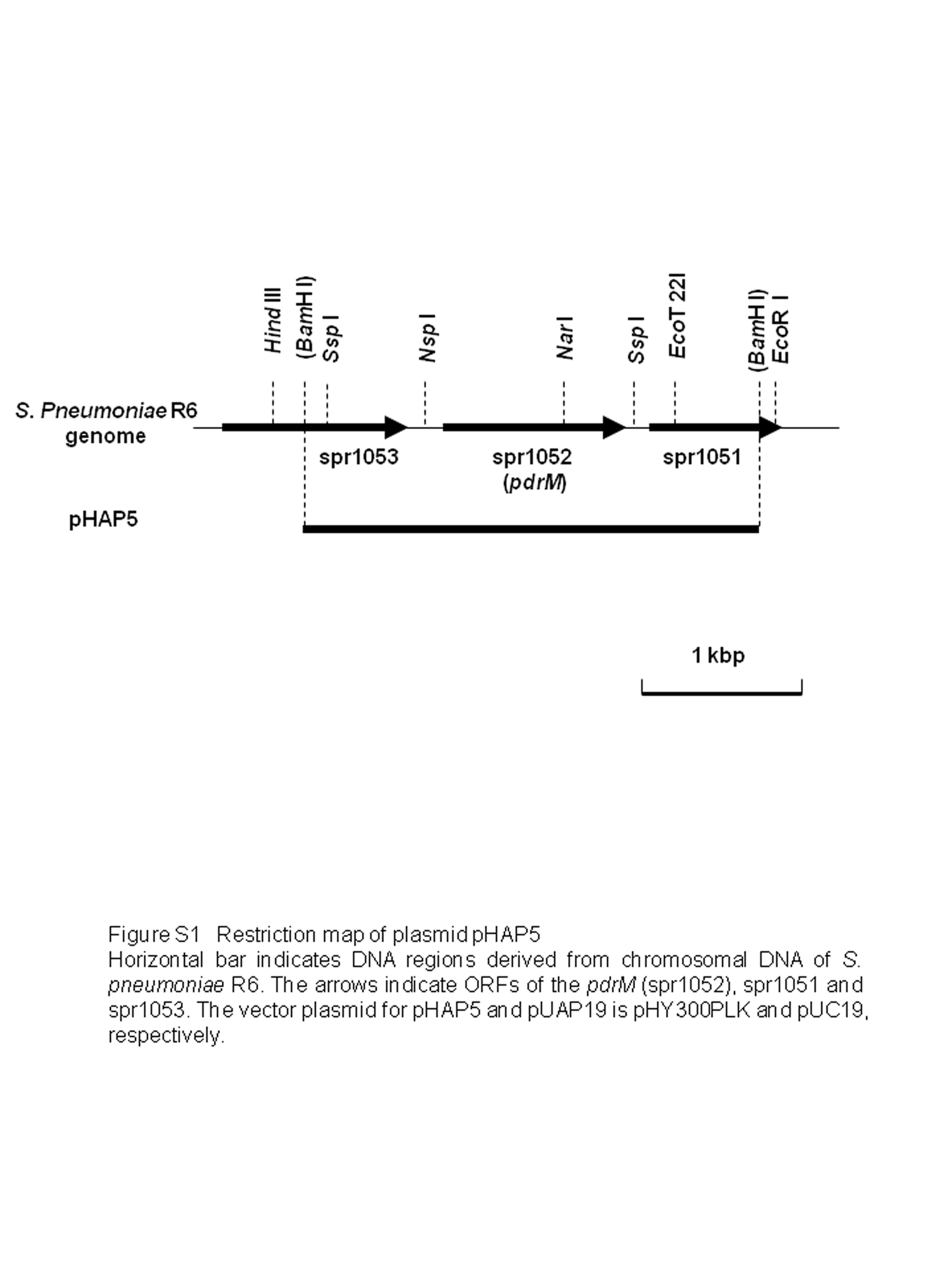

Supplement: Figure S1 — Restriction map of plasmid pHAP5. Horizontal bar indicates DNA regions derived from chromosomal DNA of S. pneumoniae R6. Arrows indicate ORFs of pdrM (spr1052), spr1051, and spr1053. The vector plasmids for pHAP5 and pUAP19 are pHY300PLK and pUC19, respectively. (TIF) [file pone.0059525.s001.tif]

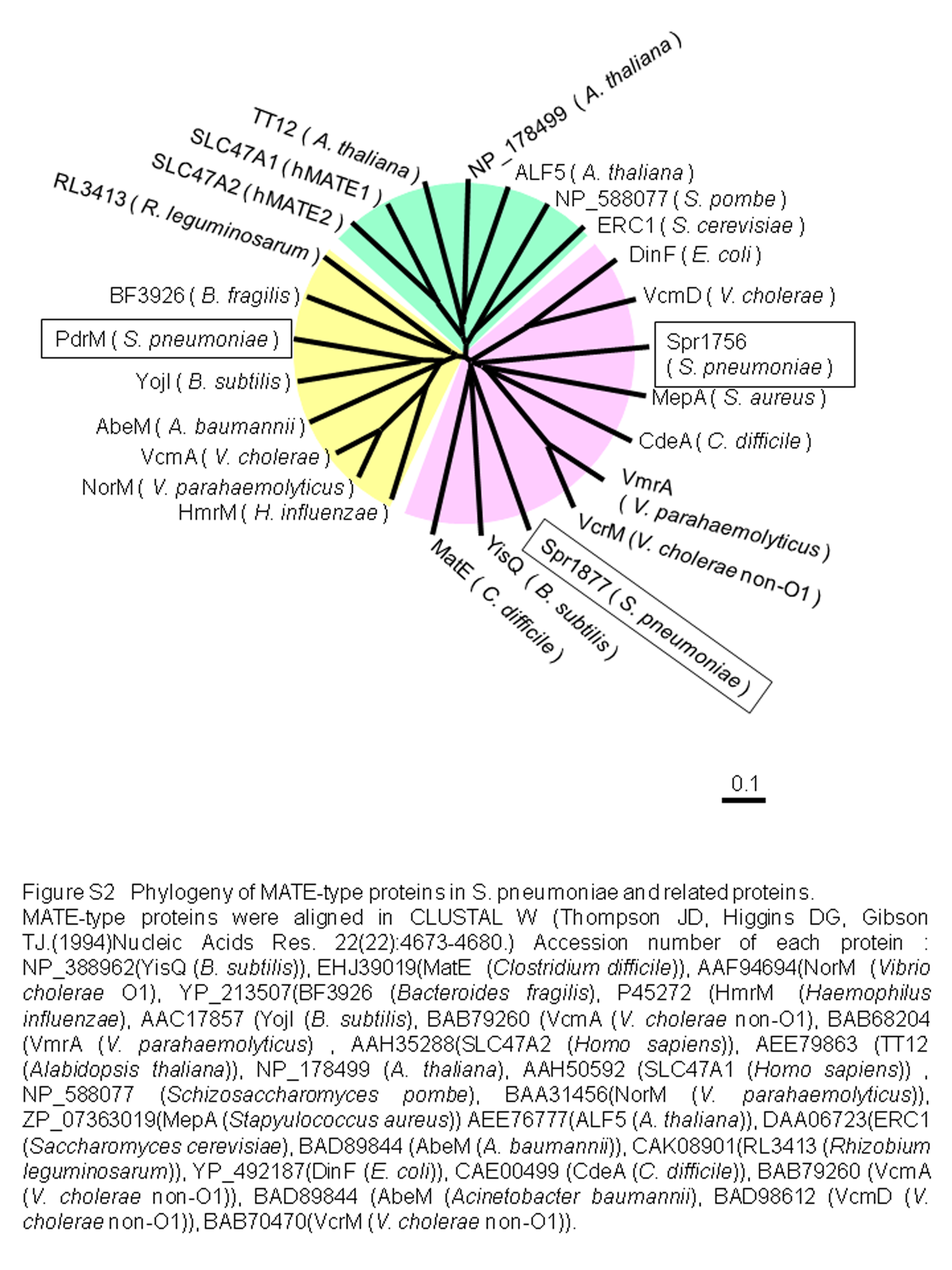

Supplement: Figure S2 — Phylogeny of MATE-type proteins in S. pneumoniae and related proteins. MATE-type proteins were aligned in CLUSTAL W (Thompson JD, Higgins DG, Gibson TJ.(1994)Nucleic Acids Res. 22(22):4673–4680.) Accession number of each protein : NP_388962(YisQ (B. subtilis)), EHJ39019(MatE (Clostridium difficile)), AAF94694(NorM (Vibrio cholerae O1), YP_213507(BF3926 (Bacteroides fragilis), P45272 (HmrM (Haemophilus influenzae), AAC17857 (YojI (B. subtilis), BAB79260 (VcmA (V. cholerae non-O1), BAB68204 (VmrA (V. parahaemolyticus), AAH35288(SLC47A2 (Homo sapiens)), AEE79863 (TT12 (Alabidopsis thaliana)), NP_178499 (A. thaliana), AAH50592 (SLC47A1 (Homo sapiens)), NP_588077 (Schizosaccharomyces pombe), BAA31456(NorM (V. parahaemolyticus)), ZP_07363019(MepA (Stapyulococcus aureus)) AEE76777(ALF5 (A. thaliana)), DAA06723(ERC1 (Saccharomyces cerevisiae), BAD89844 (AbeM (A. baumannii)), CAK08901(RL3413 (Rhizobium leguminosarum)), YP_492187(DinF (E. coli)), CAE00499 (CdeA (C. difficile)), BAB79260 (VcmA (V. cholerae non-O1)), BAD89844 (AbeM (Acinetobacter baumannii), BAD98612 (VcmD (V. cholerae non-O1)), BAB70470(VcrM (V. cholerae non-O1)). (TIF) [file pone.0059525.s002.tif]

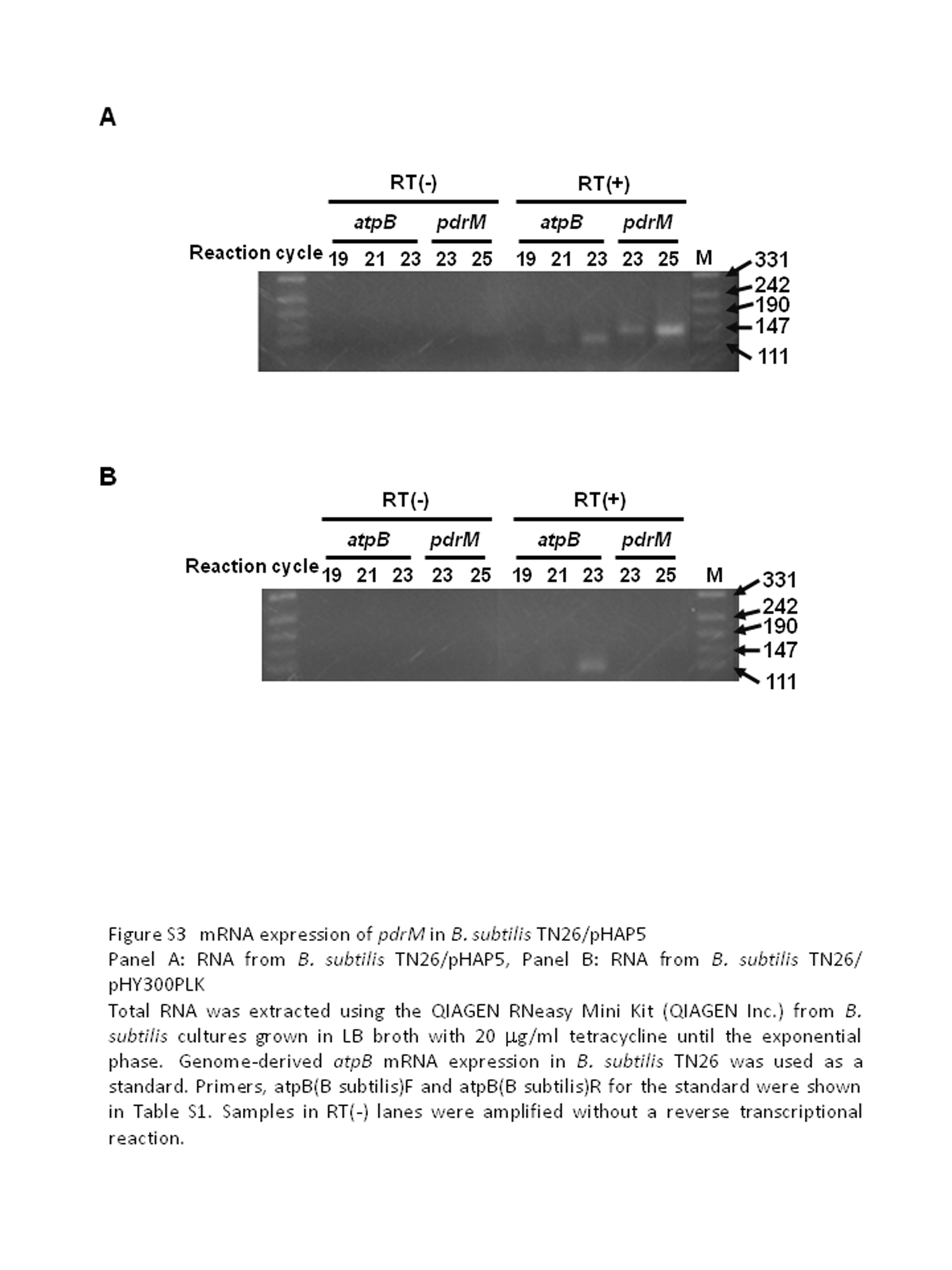

Supplement: Figure S3 — mRNA expression of pdrM in B. subtilis TN26/pHAP5. Panel A: RNA from B. subtilis TN26/pHAP5, Panel B: RNA from B. subtilis TN26/pHY300PLK Total RNA was extracted using the QIAGEN RNeasy Mini Kit (QIAGEN Inc.) from B. subtilis cultures grown in LB broth with 20 µg/ml tetracycline until the exponential phase. Genome-derived atpB mRNA expression in B. subtilis TN26 was used as a standard. Primers, atpB(B subtilis)F and atpB(B subtilis)R for the standard were shown in Table S1. Samples in RT(-) lanes were amplified without a reverse transcriptional reaction. (TIF) [file pone.0059525.s003.tif]
