# Supplementary material for: Functionally Cloned pdrM from Streptococcus pneumoniae Encodes a Na+ Coupled Multidrug Efflux Pump
Source: PLoS One. 2013 Mar 26;8(3):e59525. doi: 10.1371/journal.pone.0059525 (PMC3608713; doi:10.1371/journal.pone.0059525)
Supplement: Table S1 — Primers used in this study. (DOCX) [file pone.0059525.s004.docx]

| Table S1 Primers used in this study | | |
| --- | --- | --- |
| Primers | Sequence (5’ - 3’) | Application |
| atpB-RT-Fw | AAACGATCGATGGGACTAACTG | RT-PCR |
| atpB-RT-Rv | GACGACGAACGCTTTCTATATG | RT-PCR |
| pdrM-RT-Fw | CCCTCTCAATAGCGGATTTAAC | RT-PCR |
| pdrM-RT-Rv | AACCAGAACAGAAATCCCAAGC | RT-PCR |
| spr1756- RT-Fw | GCTTCACCTTTCCAATCTTGC | RT-PCR |
| spr1756- RT-Rv | GGTCAAAAATCGCTGTCGTC | RT-PCR |
| spr1877- RT-Fw | CTCGTTTGGTTGGTCTTGTG | RT-PCR |
| spr1877- RT-Rv | CCTCATCATAAGTCGCTCTC | RT-PCR |
| pdrM-Fw | TTTGGGATCCTATGGTCTGAAACTCTTTGG | gene cloning |
| pdrM-Rv | AATAGGATCCAAAACCCCGTGAAAACAAGG | gene cloning |
| spr1756-Fw | GGCGGATCCCTGGAAAGCTGATATAATAG | gene cloning |
| spr1756-Rv | AGGCGGATCCCTGTTAAACTATTTTCAACACC | gene cloning |
| spr1877-Fw | CATGGATCCCAGGAGGAGAGATTGTTTAAG | gene cloning |
| spr1877-Rv | GATAGGATCCAAGAGTCCAATAAAGTCCCG | gene cloning |
| PrrnO-catpt1-F | CAATGCGGAGCTTTACCTCT | amplification of Cm^r^ gene fragment |
| PrrnO-catpt1-R | CTCATATTATAAAAGCCAGTC | amplification of Cm^r^ gene fragment |
| TrpOch.F | CAGTTGCAGCCAAATTCGGC | amplification of Km^r^-Em^r^ cassette |
| trpBFdelD.r | CCTGATTTCTCGTTTCAGCG | amplification of Km^r^-Em^r^ cassette |
| bmr-UF | GCTATCAGCGCTCTAGAAAG | gene disruption(bmr) |
| bmr-UR-cat | GAAGAAGAGGTAAAGCTCCGCATTGTTACTGGAATCACAAGCCCG | gene disruption(bmr) |
| bmr-LF-cat | TAATGACTGGCTTTTATAATATGAGAGGGATTGCACTGACCATTG | gene disruption(bmr) |
| bmr-LR | ATACGCGATACACGCATACC | gene disruption(bmr) |
| bmr-UR-G | TGCAATCCCTTTACTGGAATCACAAGCCCG | gene disruption(bmr) |
| bmr-LF-G | ATTCCAGTAAAGGGATTGCACTGACCATTG | gene disruption(bmr) |
| ebrab-UF | GCTCGTTATAGGCCAGATAC | gene disruption(ebrAB) |
| ebrab-UR-cat | GAAGAAGAGGTAAAGCTCCGCATTGCGACGGAATAGACAATAACG | gene disruption(ebrAB) |
| ebrab-LF-cat | TAATGACTGGCTTTTATAATATGAGTGTATTCGGTCTCACGCTTG | gene disruption(ebrAB) |
| ebrab-LR | CGGGCGAAAATGATTGTGAG | gene disruption(ebrAB) |
| ebrab-UR-G | AATACACCTTCGAGGGAATAGCCAATAACG | gene disruption(ebrAB) |
| ebrab-LF-G | TATTCCCTCGAAGGTGTATTCGGTCTCACG | gene disruption(ebrAB) |
| blt-UF | TGCAAAGATTCCGCCTATCG | gene disruption(blt) |
| blt-UR-cat | GAAGAAGAGGTAAAGCTCCGCATTGTACACCACCCAATATCGTCG | gene disruption(blt) |
| blt-LF-cat | TAATGACTGGCTTTTATAATATGAGTCGGCCTTGCTCTTACAATG | gene disruption(blt) |
| blt-LR | GTTGGGTCCGTTATAGAAGC | gene disruption(blt) |
| blt-UR-G | GACAATCATATACACCACCCAATATCCTCG | gene disruption(blt) |
| blt-LF-G | GGGTGGTGTATATGATTGTCGGCCTTGGTC | gene disruption(blt) |
| bmrA-UF | GATCTTCAATTCCGGCTTGC | gene disruption(bmrA) |
| bmrA-UR-cat | GAAGAAGAGGTAAAGCTCCGCATTGGCCTGACAGATTTGACATAG | gene disruption(bmrA) |
| bmrA-LF-cat | TAATGACTGGCTTTTATAATATGAGCAGCAGCTGAAAATGAATGC | gene disruption(bmrA) |
| bmrA-LR | CATTGGAGAGCTGATCACTC | gene disruption(bmrA) |
| bmrA-UR-G | TCAGCTGCTGGCCTGACAGATTTGACATAG | gene disruption(bmrA) |
| bmrA-LF-G | TCTGTCAGGCCAGCAGCTGAAAATGAATGC | gene disruption(bmrA) |
| yerP-UF | ACATACCGCTCGATTCCTTC | gene disruption(yerP) |
| yerP-UR-cat | GAAGAAGAGGTAAAGCTCCGCATTGGTTTCAATATCGCCGCTGAC | gene disruption(yerP) |
| yerP-LF-cat | TAATGACTGGCTTTTATAATATGAGTTGATAAGGAAGCGGAAGGC | gene disruption(yerP) |
| yerP-LR | TGGCCGAGCATTGTTTTCAG | gene disruption(yerP) |
| yerP-UR-G | CCGATAACCGAGCTGTAGCGTGAAATCTCC | gene disruption(yerP) |
| yerP-LF-G | CGCTACAGCTCGGTTATCGGCGGTTTAATC | gene disruption(yerP) |
| bmr3-UF | AGACGTTCAAGCTCTTCCTC | gene disruption(bmr3) |
| bmr3-UR-cat | GAAGAAGAGGTAAAGCTCCGCATTGCCATAACCGCCACCATATAG | gene disruption(bmr3) |
| bmr3-LF-cat | TAATGACTGGCTTTTATAATATGAGATCTTGAACCTCGCTTTCGG | gene disruption(bmr3) |
| bmr3-LR | CAATTGGGTTAGCATTGGCG | gene disruption(bmr3) |
| bmr3-UR-G | AAGGTCATGCCCATAACCGCCACCATATAG | gene disruption(bmr3) |
| bmr3-LF-G | GCGGTTATGGGCATGACCTTAGGTTGTCAC | gene disruption(bmr3) |
| atpB(B subtilis)F | ctagccgtgatggttgttgc | RT-PCR |
| atpB(B subtilis)R | atcgggagcatgaatggaac | RT-PCR |
